# Supplementary material for: MetaRibo-Seq measures translation in microbiomes
Source: Nat Commun. 2020 Jun 29;11:3268. doi: 10.1038/s41467-020-17081-z (PMC7324362; doi:10.1038/s41467-020-17081-z)
Supplement: Supplementary file 10 — Supplementary Data 7 [file 41467_2020_17081_MOESM10_ESM.zip › File2/Confidence_VeryHigh_Taxonomy/157211_out.krona.html]

Javascript must be enabled to view this page.

members
magnitude
magnitudeUnassigned
count
unassigned
taxon
rank

157211\_out

9


SRS075341\_contig\_number\_contig-100\_9026.92119
1

1
2759
superkingdom

SRS1041145\_contig\_number\_4583
8

6
4751
kingdom


SRS098571\_contig\_number\_65123
6
1
451864
subkingdom

4890
phylum
2

2
147537
subphylum

class
4891
2

4892
order
2

4893
family
1

1
genus
71245

1

SRS058070\_contig\_number\_27677
655095
species

family
1156497
1

1
460521
genus

species
1538186
1

SRS023914\_contig\_number\_18795

3
phylum
5204

5302
subphylum
3

3
class
155616

order
1851469
3

1759442
family
3

3
5552
genus

species
82510
3

SRS013687\_contig\_number\_20231SRS051031\_contig\_number\_contig-100\_2751.168984SRS053356\_contig\_number\_16236

1
33208
kingdom

1
phylum
7711

89593
subphylum
1

superclass
8287
1

class
40674
1

superorder
314146
1

1
order
9443

1
376913
suborder

314293
infraorder
1

parvorder
9526
1

1
314295
superfamily

9604
family
1

1
207598
subfamily

genus
9605
1

9606
species
1

SRS076929\_contig\_number\_contig-100\_44.121119
